# Neuroschistosomiasis mimicking lower back pain: case report of a rare differential diagnosis in a pediatric patient

**DOI:** 10.1186/s13037-018-0175-z

**Published:** 2018-10-06

**Authors:** Abdulrahman Hamad Al-Abdulwahhab, Abdulaziz Mohammad Al-Sharydah, Sari Saleh Al-Suhibani, Saeed Ahmad Al-Jubran, Ali Khalaf Al-Haidey, Abdulkhaliq Ibrahim Al-Hifzi, Wissam Al-Issawi

**Affiliations:** 1 Radiology Department, King Fahd Hospital of the University, Imam Abdulrahman Bin Faisal University, P.O. box: 4398, Khobar City, Eastern Province 31952 Saudi Arabia; 2 Radiology Department, Prince Sultan Military Medical City, Riyadh, Al-Riyadh Province Saudi Arabia; 3 Neurosurgery Department, King Fahd Hospital of the University, Imam Abdulrahman Bin Faisal University, Khobar City, Eastern Province 31952 Saudi Arabia

**Keywords:** Conus medullaris, Magnetic resonance imaging, Myelitis, Neuroschistosomiasis

## Abstract

**Background:**

Spinal myelitis is an infrequent manifestation of spinal cord infection. It is caused by the *Schistosoma* species, which are endemic in South America, part of the Middle East, and Africa.

**Case presentation:**

We report the case of a 13-year-old male adolescent complaining of progressive lower back pain and weakness of the lower extremities for 3 days. Initial magnetic resonance imaging revealed typical transverse myelitis. Subsequently, parasite serology showed a markedly elevated level of *Schistosoma* antibody titers, and cerebrospinal fluid analysis yielded normal results. Because of our presumptive diagnosis of neuroschistosomiasis, the patient was prescribed an empirical regimen of an anti-parasitic agent, after which his neurological deficit promptly subsided. The patient was followed for 1 year and showed a complete long-term resolution of symptoms.

**Conclusions:**

This case highlights the increasing prevalence of neuroschistosomiasis in recent years, particularly in patients with a history of travel to endemic regions. Moreover, the study reports the clinicoradiological features of this enigmatic disorder. This rare occurrence potentiates further studies to address unanswered questions about neuroschistosomiasis.

## Background

Schistosomiasis or bilharziasis is a blood-dwelling fluke disease caused by a trematode worm of five organisms of a species, namely *Schistosoma haematobium*, *S. mansoni*, *S. japonicum*, *S. guineensis*, and *S*. *mekongi*. It causes various gastrointestinal or urogenital tract manifestations [1].

Schistosomiasis, considered one of the most common human parasitic infections that affects the middle eastern regions, particularly the southern region of Saudi Arabia, is commonly caused by *S*. *haematobium* and *S. mansoni* [2]. Despite schistosomiasis being endemic to Saudi Arabia, the incidence of neuroschistosomiasis is rarely reported [3].

Typically, schistosomiasis symptoms are nonspecific and depend on the type of *Schistosoma* species affecting the host, as well as the stage of the cycle (acute vs. chronic). Patients with cerebral involvements may complain of a headache, seizures, hydrocephalus, increased intracranial pressure, and focal neurological deficits. However, spinal schistosomiasis symptoms range from radiculopathy to myelopathy in different clinical settings, such as lower limb weakness, paresthesia, deep tendon reflex abnormalities, spastic paraplegia, cauda equina syndrome, and bladder dysfunction [4].

Notably, the predominant spinal complaint is lower back pain radiating to the lower limbs [4]. In this study, we present a rare clinical scenario of neuroschistosomiasis as a cause of back pain in a pediatric patient, with its clinico-radiological characteristics in concordance with CARE guidelines.

## Case presentation

A 13-year-old male adolescent, with no history of any medical illnesses, presented to the emergency room complaining of severe continuous backache and fatigability for 3 days. He had recently traveled to the southern region of Saudi Arabia. No bowel or bladder symptoms were present. Written informed consent was obtained from the patient by King Fahd Hospital of the University, Imam Abdulrahman Bin Faisal University.

Clinical examination revealed tenderness of the lower back region on palpation and a reduction in the strength of both knees and hip during extension and flexion (grade 3/5), with sensory loss in both lower limbs on pinprick examination. Other parameters on neurological examination were intact. Laboratory results revealed anemia with mild leukocytosis and peripheral eosinophilia. All other results of routine laboratory tests were within the reference range.

**Fig. 1:**
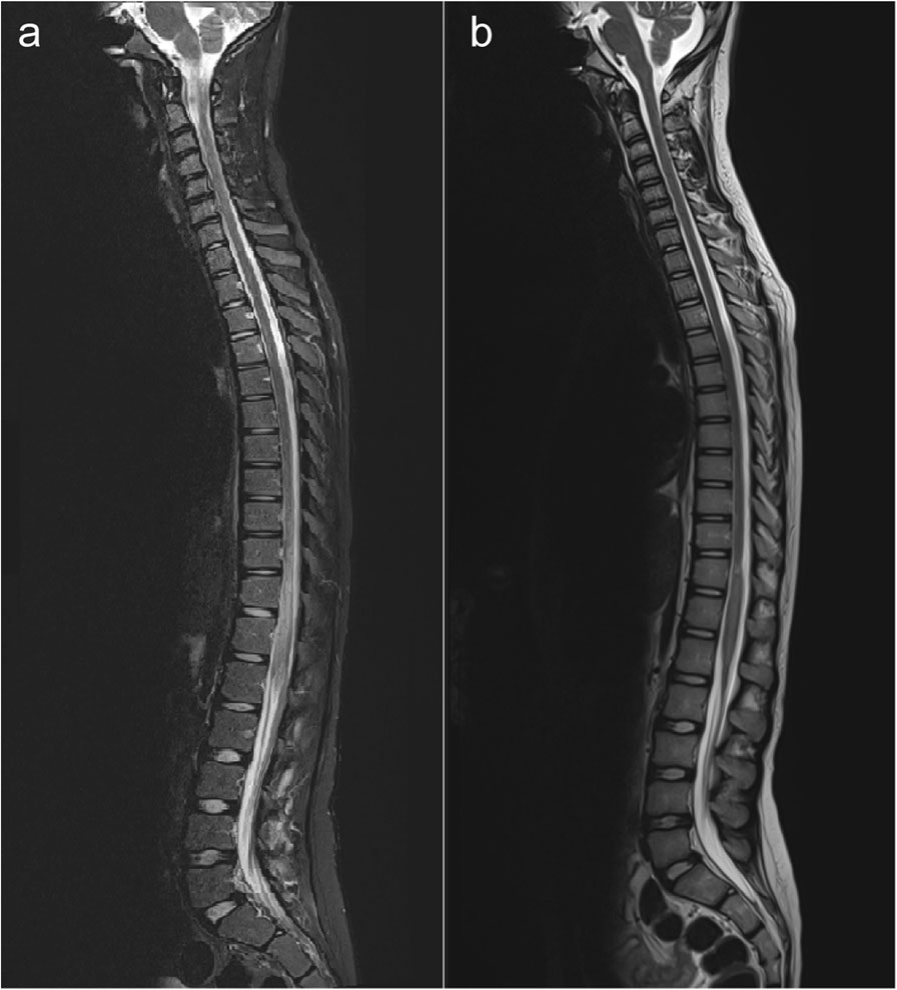
Demonstration of spinal cord schistosomiasis pre- and post-treatment. **a** Pre-praziquantel treatment. Sagittal T2-weighted magnetic resonance imaging revealed a circumferential and uniformly enlarged caudal spinal cord, including the thoracolumbar spine, in addition to a hyperintense signal intensity relative to the normal appearance of the cranial part of the spinal cord. **b** Post-praziquantel treatment. Sagittal T2-weighted magnetic resonance imaging revealed a significant regression of cord enlargement as well as high signal intensity in the caudal spinal cord

**Fig. 2:**
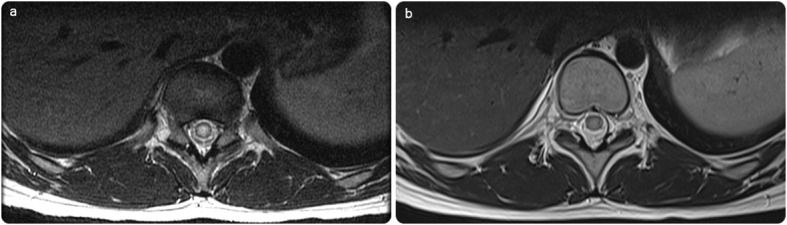
**a** Initial axial T2 of the dorsolumbar spine shows intramedullary expansion with T2 hyperintensity. **b** Significant regression in the follow-up image post-praziquantel treatment, with normalizing signal of the spinal cord

An X-ray examination of the lumbar spine showed no gross abnormality. Emergent magnetic resonance imaging (MRI) revealed cord edema with an abnormal signal intensity in the thoracic and lumbar regions (Figs. 1a, 2a). The clinical and laboratory findings of the radiological features indicated acute transverse myelitis secondary to infectious or inflammatory changes. However, the possibility of other differentials remained. A lumbar puncture was performed using standard procedures. Gram staining and culture of the cerebrospinal fluid yielded negative results. No isolated parasitic eggs were present in the urine or stool specimens. Brain MRI findings were unremarkable. However, the *Schistosoma* serology titer showed a marked elevation.

Therefore, a presumptive diagnosis of neuroschistosomiasis was made, and an experimental oral treatment for schistosomiasis was prescribed without any spinal intervention. The patient’s symptoms and signs rapidly subsided, with a regression of the spinal myelitis pattern on follow-up MRI examination (Figs. 1b, 2b). Subsequently, he was discharged from the hospital within 1 week in dependable health and continued his anti-microbial regimen for 1 month. Follow-up examinations at the neurology clinic revealed a gradual improvement in the patient’s clinical condition. The patient was referred for further follow-up in the infection clinic. The patient was followed for 1 year and showed a complete long-term resolution of symptoms.

## Discussion

Neuroschistosomiasis is caused by *Schistosoma* species that infect the central nervous system, either the brain or the spinal cord. This clinical condition is often underdiagnosed and under-recognized with a high risk of disability if not treated early [4]. The first case of neuroschistosomiasis was reported by Coyle et al. in a German traveler who developed spinal myelitis after visiting Brazil in 1930 [5]. The involvement of the central nervous system is rare among *Schistosoma* species (*S. japonicum* is the most common species), which affects the brain and induces cerebral encephalitis. In contrast, *S. haematobium* and *S. mansoni* are considered the most common species that affect the spinal column, with clinical presentations ranging from radiculopathy to myelitis [6]. Globally, *S. mansoni* is the most common *Schistosoma* species affecting the spinal cord [7].

*Schistosoma* species spread in the central nervous system when mature sexual worms or parasitic eggs travel through the retrograde pelvis venous flow into the venous plexus of vertebral and epidural Batson veins [4]. The preferential location of spinal schistosomiasis is in the lower spinal cord, likely related to the anastomotic site between the Batson venous plexus and pelvic veins located between T11 and L1 levels [8].

Spinal schistosomiasis has four standard clinicopathological features: granulomatous, radicular, myelitis, or vascular form. The *Schistosoma* eggs tend to release many proteolytic enzymes that induce a local eosinophilic inflammation and aggregate cytokines to perform a granulomatous mass over time [9].

The appropriate diagnosis of neuroschistosomiasis is achieved by a combination of clinical history, laboratory investigation, and neurodiagnostic imaging. However, the histopathological result of the nervous tissue is the only definitive component for making a diagnosis and considers the presence of parasitic eggs with granulomatous changes in biopsy specimens [10]. However, it is considered an invasive procedure and can lead to compromised neurological function.

**Fig. 3:**
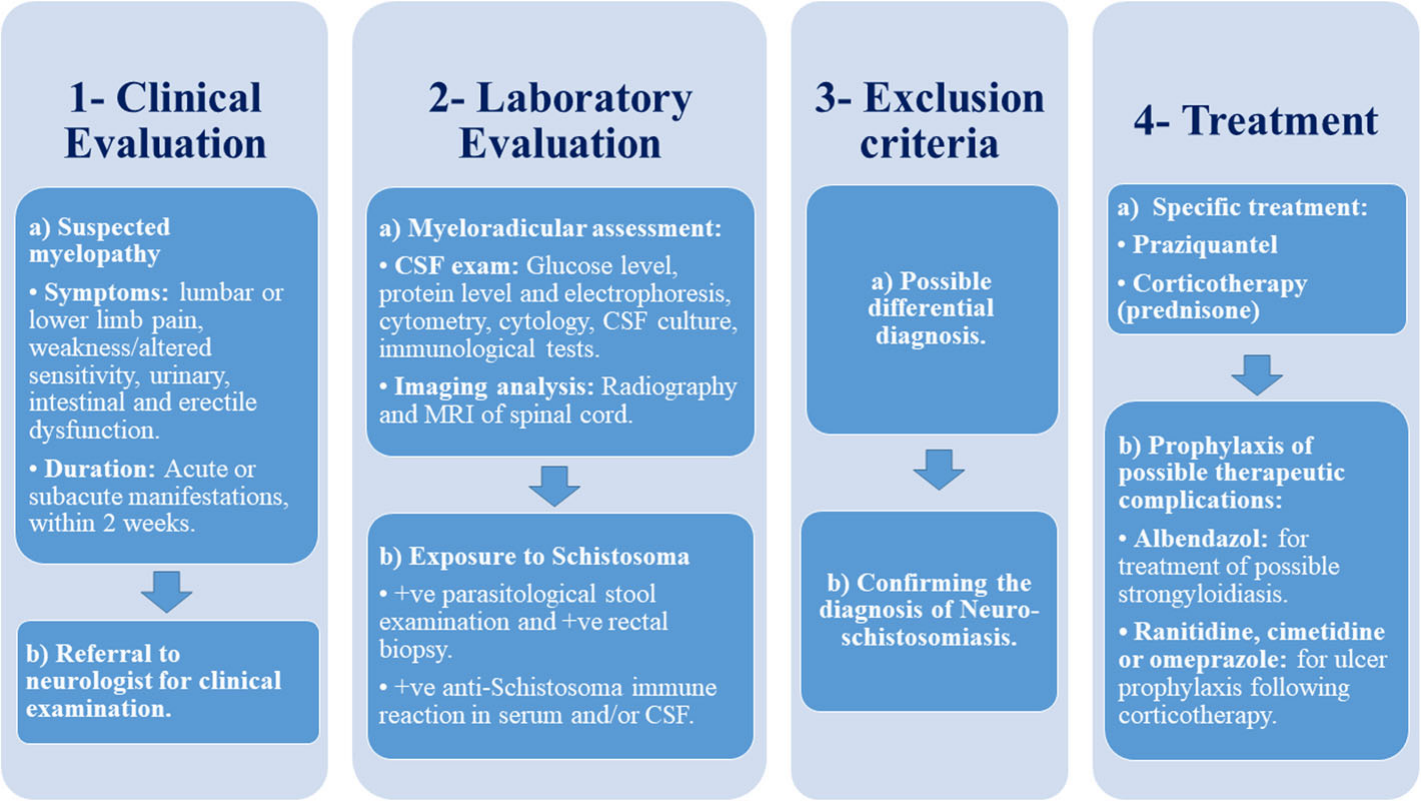
Algorithm for the systematic diagnosis of neuroschistosomiasis, without an invasive biopsy, and a brief discussion of treatment

In our pediatric case, we administered empirical anti-parasitic therapy initially to preserve our conservative treatment and to further evaluate whether a surgical biopsy is needed. Our case responded promptly, with a significant resolution of the symptoms. We believe that in the absence of a histological diagnosis, a presumptive diagnosis of spinal schistosomiasis can be established by a mindful interpretation of laboratory results through the presence of positive parasitological exposure to the host through urine and stool screening for *Schistosoma* eggs or a rectal biopsy, which is considered more sensitive for *S. mansoni* and exclusion of other cases of spinal myelitis [4]. Nascimento-Carvalho et al. conducted a retrospective study of 73 patients (age: < 20 years) and reported that in places where *Schistosoma* infection is common, neuroschistosomiasis should be diagnosed presumptively with particular attention to pediatric patients manifesting neurological signs and symptoms [9]. Hence, based on our literature review and personal experience at our academic institution, we propose a diagnostic algorithm for diagnosing neuroschistosomiasis and to obviate the need for obtaining an invasive biopsy in the (Fig. 3) provided.

The radiological assessment of the spine can play a significant role in detecting the disease location and evaluate the patterns through MRI, and it yields variable imaging findings, including acute spinal myelitis and spinal cord compression secondary to an extramedullary granulomatous mass or a focal intramedullary granulomatous mass. Nevertheless, the most frequently encountered abnormalities, as observed in our patient, are intramedullary, expanding in the caudal spinal cord because of acute spinal myelitis [11].

The antibody assay is a sensitive diagnostic technique for schistosomiasis, but is still limited to differentiate between an active and chronic form of *Schistosoma* exposure. New diagnostic tools for neuroschistosomiasis have only evolved a couple of years ago, particularly with the development of polymerase chain reaction to amplify DNA revolutionized clinical medicine. These new techniques analyze the DNA of *Schistosoma* infectious agents in the cerebrospinal fluid of patients. Moreover, these techniques seem to implicate high specificity (100%) and sensitivity (80%) compared with our standard laboratory tests. Only a few studies have evaluated this method, and future studies with a high quality of methodical designing are warranted [12].

A prompt management of spinal schistosomiasis is crucial to improve the chances of neurological recovery. Randomized controlled trials have shown that an anti-parasitic drug, praziquantel, is an effective oral drug against all *Schistosoma* species. Corticosteroids may be needed as adjuvants to praziquantel in neuroschistosomiasis to minimize the allergic reactions and reduce the expected complications [13].

## Conclusions

The involvement of the central nervous system is a rare complication of schistosomiasis. The presumptive diagnosis of neuroschistosomiasis should be considered when investigating a case of vague back pain, particularly in pediatric patients with a history of recent travel to endemic regions and an abnormal *Schistosoma* serology. It is imperative to initiate anti-parasitic medication at the earliest to prevent severe neurological sequela. Newly developed DNA diagnostic techniques of neuroschistosomiasis are promising and mandate further assessment of unanswered questions.

## Abbreviation

MRI: Magnetic resonance imaging
